# Effect of contact with podiatry in a team approach context on diabetic foot ulcer and lower extremity amputation: systematic review and meta-analysis

**DOI:** 10.1186/s13047-020-0380-8

**Published:** 2020-03-20

**Authors:** Virginie Blanchette, Magali Brousseau-Foley, Lyne Cloutier

**Affiliations:** 1 Department of Human Kinetic and Podiatric Medicine, Université du Québec à Trois-Rivières, 3351, boul. des Forges, C.P. 500, Trois-Rivières, Québec, G9A 5H7 Canada; 2 Centre intégré universitaire de santé et de services sociaux de la Mauricie-et-du-Centre-du-Québec (CIUSSS-MCQ) affiliated to Université de Montréal, Faculty of Medicine, Family Medicine Unit, Trois-Rivières, Québec, G9A 1X9 Canada; 3 Department of Nursing, Université du Québec à Trois-Rivières, 3351, boul. des Forges, C.P. 500, Trois-Rivières, Québec, G9A 5H7 Canada

**Keywords:** Systematic review, Diabetes, Multidisciplinary team, Podiatry, Foot ulceration, Amputation

## Abstract

Multidisciplinary team (MDT) approach has been shown to reduce diabetic foot ulcerations (DFUs) and lower extremity amputations (LEAs), but there is heterogeneity between team members and interventions. Podiatrists have been suggested as “gatekeepers” for the prevention and management of DFUs. The purpose of our study is to review the effect of podiatric interventions in MDTs on DFUs and LEAs. We conducted a systematic review of available literature. Data’s heterogeneity about DFU outcomes made it impossible for us to include it in a meta-analysis, but we identified 12 studies fulfilling inclusion criteria that allowed for them to be included for LEA outcomes. With the exception of one study, all reported favourable outcomes for MDTs that include podiatry. We found statistical significance in favour of an MDT approach including podiatrists for our primary outcome (total LEAs (RR: 0.69, 95% CI 0.54–0.89, I^2^ = 64%, *P* = 0.002)) and major LEAs (RR: 0.45, 95% CI 0.23–0.90, I^2^ = 67%, *P* < 0.02). Our systematic review, with a standard search strategy, is the first to specifically address the relevant role of podiatrists and their interventions in an MDT approach for DFU management. Our observations support the literature that MDTs including podiatrists have a positive effect on patient outcomes but there is insufficient evidence that MDTs with podiatry management can reduce the risk of LEAs. Our study highlights the necessity for intervention descriptions and role definition in team approach in daily practice and in published literature.

## Introduction

Diabetes is a worldwide health issue and of its many complications, diabetic foot ulceration (DFU) is a prominent problem [1]. Up to 25% of people with diabetes will experience a DFU in their lifetime, and about 85% of lower limb amputations are preceded by a DFU [2, 3]. The 5-year mortality rate exceeds 70% with a lower-extremity amputation (LEA) [4, 5]. Consequently, DFUs in diabetes patients should be perceived as a major warning sign for morbidity and mortality, and as such, they require close monitoring, medical follow-up, and integrated foot care [6, 7]. Integrated foot care is a pathway of care management with rapid and appropriate access to a multidisciplinary team (MDT) for coordinated care between hospital and community services [8]. An MDT in which health professionals work together to achieve the best outcomes for patients with an at-risk diabetic foot has been developed in response to the need for improved methods of service delivery. A number of health disciplines can be involved such as medicine (general medicine, endocrinology, infectious diseases medicine, and vascular, plastic and orthopaedic surgeries), podiatry, nursing, nutrition, orthotics and prosthetics, physiotherapy, and psychology. Each discipline’s implication in the MDT depends on the stage of the DFU, but podiatry has a central role throughout [9–11]. The first foot care MDTs were established in the United Kingdom in the late 1980s and highlighted the role of chiropody (former designation for podiatry).

Previous systematic reviews have linked MDTs to lower LEA rates following a DFU, noting the high heterogeneity of MDTs composition and interventions [12–15]. Neither of these reviews, however, looked specifically at a specific member and its interventions. International guidelines recommend at least 3 levels of foot care management based on foot risk, and podiatrists are included at each level [16]. Many studies about multidisciplinary healthcare centres in Europe and in the United States reported that this approach helped reduce amputation rates by 36 to 86% [17, 18]. However, the structures and delivery of these MDTs vary across settings and countries [19–22]. As part of an MDT approach, podiatrists have been suggested to serve as “gatekeepers” for the prevention and management of diabetes-related foot complications [11, 23]. Podiatric management of an at-risk diabetic foot has an underlying focus on preventive screening, education, offloading, and foot care [2]. It is usually assumed that podiatry prevents LEA; however there is insufficient evidence to demonstrate the effect of patient contact with a podiatrist in a systematic review and meta-analysis [24]. So far, the effects of MDTs including podiatry and its interventions have not been demonstrated [12, 13, 24]. The partnership with podiatrists for DFU management in MDTs is a logical one, and the expertise and skills of each team member can improve outcomes and limb salvage [25]. It is well known that podiatry is limited and is a variable resource (in terms of accessibility, financial coverage and scope of practice) in several healthcare systems. In that context, it is sensible to question whether or the podiatrist is a resource that makes a difference for patient outcomes. There is therefore a need to look for the effectiveness of MDTs which specifically include contact with podiatry (from different scope of practice around the world) on DFUs and LEAs in people with diabetes. The objective of this study is to examine the effect of patients’ contact with podiatry in MDTs and highlight its specific role and, if possible, determine which podiatric interventions play a key role in MDTs.

## Method

### exclusion criteria/exclusion criteria

The research question was “What is the effect of contact with a podiatrist and their interventions in an MDT context, on LEAs and DFUs, in individuals with diabetes?” [26]. Results for the effect of podiatric interventions without an MDT with a similar methodological approach will be presented in a different article. Inclusion criteria were studies that included participants 18 years of age or older and having a diagnosis of either type 1 or 2 diabetes. There were no restrictions regarding the date of publication, geographical location, or study setting. Randomised controlled trials (RCTs), cohorts, either prospective or retrospective, and comparative cohorts before and after for which a reported effect on LEAs or on DFUs was available were included. The targeted interventions (presented in Table 1) were educational prevention, foot cares, offloading, infection control, wound cares and surgical strategies that had to be specifically delivered by a podiatrist in a multidisciplinary context or in an MDT program. The participation of the podiatrist had to be clearly identified in the article. To be included, articles had to use a comparison group including interventions or treatments without an MDT context. Potential measured outcomes that were deemed relevant for this study are presented in Table 2. The exclusion criteria for the articles were: patients with gestational diabetes, language other than French and English, and type of publications such as case-control studies, cross-sectional studies, audit, review articles, charts reviews, cases series, and case studies, as well as conference and communication papers. Finally, a predefined review protocol was registered at the PROSPERO international prospective register of systematic reviews, registration number CRD42017057851 [28].

**Table 1:** Podiatric interventions

Categories [16, 27]	Examples of podiatric interventions
Preventive strategies	- Stratification of the population risk - Program for vulnerable populations - Pedorthic evaluation
Educational strategies	- Program for self-management and support for self-management - Personal hygiene education
Foot cares strategies	- Callus management - Nail management
Offloading strategies	- Orthoses - Management with shoes - Walking aids - Immobilisation
Infection control and wound care strategies	- Specialised wound dressing - Infection algorithm - Biofilm-based wound care - Advanced adjuvant therapies such as hyperbaric oxygen therapy, negative pressure therapy, etc.
Surgical strategies	- Surgical debridement - Correction of bone deformities - Tissue engineering and grafts
Other strategies from podiatric expertise	- Pharmacology - Radiology

**Table 2:** List of potential outcomes measured

Outcomes			
**Primary**	DFUs	Prevent	• Rate • Frequency • Prevalence • Incidence • Data about wound healing
		Improve	
		Cure	
	LEAs	Prevent	• Limb salvage
		Improve	• Rates • **Level of LEAs** • Ratio (high-low) • **Frequency** • Prevalence • Incidence • Time to amputation
**Secondary**	Mortality/survival		
	Recurrence	• DFUs	
		• LEAs • Reamputation	
	Other complications	• Infection • Other foot problems	
	Healthcare data	Utilization of resources	• Hospitalisation (number of admissions) • Length of hospital stay • Cost-effectiveness
	Patient satisfaction		

**Bold characters**: Outcomes included in meta-analysis

### Search strategy

CENTRAL, CINAHL, EMBASE and MEDLINE databases were searched to identify relevant studies published up to February 1, 2020. The strategy was adapted as per database requirements, and we combined the results from the different databases and are available in Additional file 1. We also searched for other potential publications identified through search strategy from grey literature and references cited in relevant articles [29].

### Data collection, extraction and management

Two review authors (VB and MBF) independently assessed the titles and abstracts of all studies obtained from the databases, and full copies of the articles that met the inclusion criteria were consulted for the next step. In case of disagreement or doubt between the two authors, a decision was obtained by consensus following a discussion between reviewers (VB and MBF). If a consensus could not be reached, a third reviewer was consulted (LC). Following the selection, the Cohen’s kappa was calculated to measure the agreement between the two independent authors.

Data from included articles was extracted and recorded independently by two review authors (VB and MBF) using a standardised extraction sheet adapted for the data of this review [26]. Data sheets were compared and discrepancies were discussed between the two investigators (VB and MBF). Risk of foot disease at baseline was assessed using the Diabetic foot risk stratification and triage system from the SIGN (Scottish Intercollegiate Guidelines Network) system guidelines because this system showed higher diagnostic accuracy values [30, 31]. If the data required was missing from the published article, we tried to reach the authors.

### Assessment of risk of bias in included studies

Assessment of risk of bias was dependent on each study’s design. For cohort and pre and post cohorts, *The Joanna Briggs Institute Critical Appraisal Tools for Systematic Reviews* were performed through a qualitative evaluation checklist specifically elaborated for these designs [32]. Results are expressed by the frequency of each classification. Risk of bias assessment was performed for within and across studies independently by the two authors (VB and MBF). A third reviewer (LC) was involved to resolve disagreements. Excel (Microsoft Corporation, Redmond, WA, USA) was used to represent the potential risk of bias.

### Measures of treatment effects and synthesis

When appropriate, meta-analysis was performed in order to pool outcome data with Review Manager version 5.3 (RevMan, The Cochrane Collaboration, Oxford, United Kingdom) for statistical analysis for suitable studies [33]. We also assessed the heterogeneity by using the Cochrane’s Q statistic (I^2^ index). Quantitative synthesis using the Mantel-Haenszel method with fixed effect models (I^2^ index inferior or equal to 50%) or random effect models (I^2^ index between 50 and 75%) were used. We considered an I^2^ index greater than 75% indicative of substantial statistical heterogeneity [34]. In such cases, a qualitative analysis and narrative synthesis were produced. Risk ratios (RR) were chosen for reporting the pooled effect of dichotomous data with a confidence interval (CI) of 95%. Generic effect of inverse variance model was used when studies reported association measures. Statistical significance was set at *p* < 0.05. To assess publication bias, a funnel plot of the overall estimate of a primary outcome and its standard error (SE) was performed.

### Subgroup analysis

We decided to analyse whether the role of podiatrists in the MDT with regard to their implication in the team was primary (leading role or core of the team), secondary (support to the MDT but not the leading role), or tertiary (external consultation when needed). We also conducted different subgroup analyses based on our predetermined outcomes such as risk stratification of the population, healthcare setting, quality of studies, comorbidities and risk factors, types of wound (neuropathic, ischemic), etc.

## Results

### Literature search

**Fig. 1:**
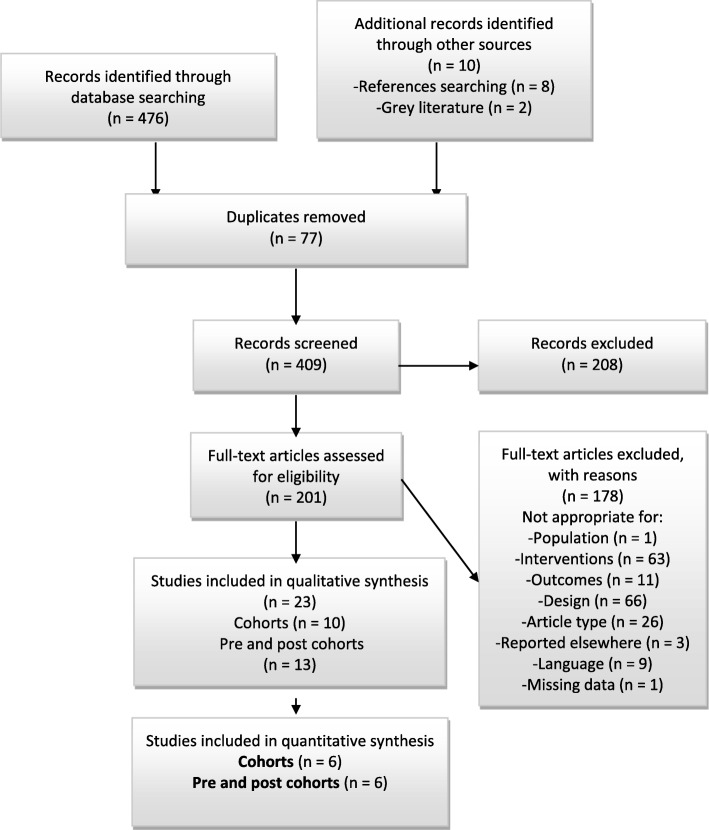
PRIMA flow diagram

From 4987 titles retrieved from the databases, 2 from grey literature, and another 10 from reference lists of potential included studies (see Additional file 3), 476 articles were reviewed for titles and abstract after removing duplicates. Following this selection, the Cohen’s kappa was calculated between the two independent authors (VB and MBF) and was of 0.96, indicating excellent agreement between both reviewers. We then identified 26 articles that met the eligibility criteria (10 cohorts, 16 comparative pre and post cohorts, and 0 RCTs). None of studies from the grey literature or reference lists were included.. A PRISMA flow diagram with motives for exclusion of 178 studies is represented in Fig. 1, and details of excluded studies are in Additional file 4. Twenty-six studies that reported outcomes for podiatric interventions in an MDT context were included in this systematic review. Of these, 3 sets of articles were from the same group of authors, [35–38] and [39, 40]. The decision was made to exclude the oldest ones, based on the fact that the same data set was used. Therefore, 23 studies were included in this systematic review and only 12 for the meta-analysis, considering that 11 studies did not meet the eligibility criteria after full-text reading and analysis. One included cohort study had 4 substudies [41] and another, 2 substudies [38]. Reasons for exclusion (consensus between authors) were: mixed data when reporting primary outcome [42], eminent difference of basic population [43–45], podiatric interventions pre and post cohort [46] and incomplete data for pooling the outcomes [40, 47–51].

### Description of included articles

Characteristics of the studies included for meta-analysis (*n* = 12), such as study design and information concerning length of follow-up, setting, source of data, participants, interventions and description of the MDT, comparison, outcomes, and risk stratification are presented in Additional file 5. All 23 studies included in the systematic review were in English. We identified 6 studies from the United States [40, 45, 47, 48, 54, 55], 2 from Canada [44, 55] and 10 from Europe, of which 5 were from the United Kingdom [41, 43, 50, 56, 57], 2 from Spain [36, 38], 1 from Sweden [51], 1 from the Netherlands [58] and 1 from Italy [49]. There were also 3 articles from Asia, of which 2 were from China [42, 59] and 1 from Singapore [60]. One publication was respectively from Australia [61] and another from New Zealand [46]. Publication years were from 1990 to 2019. Four articles were published before 2000 [50–52, 57], and 3 articles were from 2000 to 2009 [38, 43, 49], while the majority (16 articles) was published between 2010 and 2019 [36, 40–42, 45–47, 54–56, 58, 60–63]. Lengths of follow-ups were between 1 and 14 years, with a median of 3.8 years and a mean of 3.6 years. Study settings were mostly in tertiary care [36, 38, 40, 43–47, 50, 54, 55, 57–59, 61]. There were 4 studies based in primary care [42, 48, 49, 63], 3 in secondary care settings [51, 56, 60] and 1 unknown [41]. Three articles collected prospective data [38, 50, 57]; all other analyses were carried out using retrospective data (electronic medical records, medical charts, databases with coding). The 12 articles which were combined for meta-analysis accounted for 545,829 patients. The participants’ characteristics at baseline were heterogeneous. According to our stratification system of choice for the population (SIGN) [30], 21 studies had a population stratification categorised as high risk. This is explained by the fact that the population included in the studies could either have a DFU or a history of DFU [45, 47, 50, 55, 57, 58, 60, 61], an amputation or a history of amputation [36, 40, 43–45, 48, 51, 61], peripheral vascular disease (PVD) [45, 56], or diabetic foot infection [52, 61, 62]. Stratification of the population with PVD, neuropathy, cellulitis, osteomyelitis or Charcot foot is also categorized as a moderate to high-risk population [41]. Four articles included both categories (high and low risk) [38, 42, 49, 54] and 4 articles had a system of classification of their population or DFUs: surgery classification [47], LEAs risk with King’s classification [60], Wagner’s classification for ulcers [59], and Texas University classification for DFUs [54].

The specific podiatric interventions were all poorly described (without information concerning nature, intensity, duration, frequency) and very heterogeneous. In the 12 included studies, podiatric interventions are stated as contact with podiatry [36, 40–42, 45, 49, 51, 55, 58, 59, 61, 63]. Thus, we classified the podiatric interventions as educational strategies [38, 43, 50, 54, 57, 60], foot care strategies [38, 43, 46, 50, 54, 56, 57, 60], offloading strategies [43, 46, 48, 55–57], wound care and infection control strategies [44, 48, 54], surgical strategies [44, 47, 54], and stratification [38, 42, 49]. Only a few studies had defined exposure to the interventions as a weekly exposure to podiatry [56, 60], a regular follow-up in podiatry or monthly appointments [38, 43, 50] or at least every 3 months [57]. Concerning the role of the podiatrist, we decided a posteriori to distinguish their role according to their implication in the MDT. With this in mind, the podiatrist intervenes in a primary role in 8 articles (leading role or core of the team) [36, 43, 44, 47, 48, 54, 55, 59]. Specifically, in these articles, the podiatrist formed the core of the team with endocrinologists [36, 59], nurses [43, 55], and vascular surgeons [44, 47, 54]. Podiatrists are sole leaders in one article [48]. In 8 articles, they had a secondary role (support to the MDT but without a leading role) [45, 46, 49, 51, 56, 58, 60, 61] and in 2 articles, they had a tertiary role (external consultation when needed) [38, 42]. Podiatrists’ role was similar to other team members in two articles [50, 57] . Finally, in 3 articles, it was impossible to determine the level of the podiatrist’s implication in the MDT because no description of the team was provided. In one article [52], it was a podiatry-established critical pathway and in the two others, it was with other lower-extremity specialists [40, 41]. The MDTs composition was also variable; some MDTs showed care management in 2 levels of team members’ implication [36, 42, 47, 49]. Finally, funding and conflict of interest in the included articles were clearly mentioned in the full text of 14 out of 23 articles [36, 41–44, 47, 48, 51, 54, 55, 57, 60–62].

### Primary outcomes

**Fig. 2:**
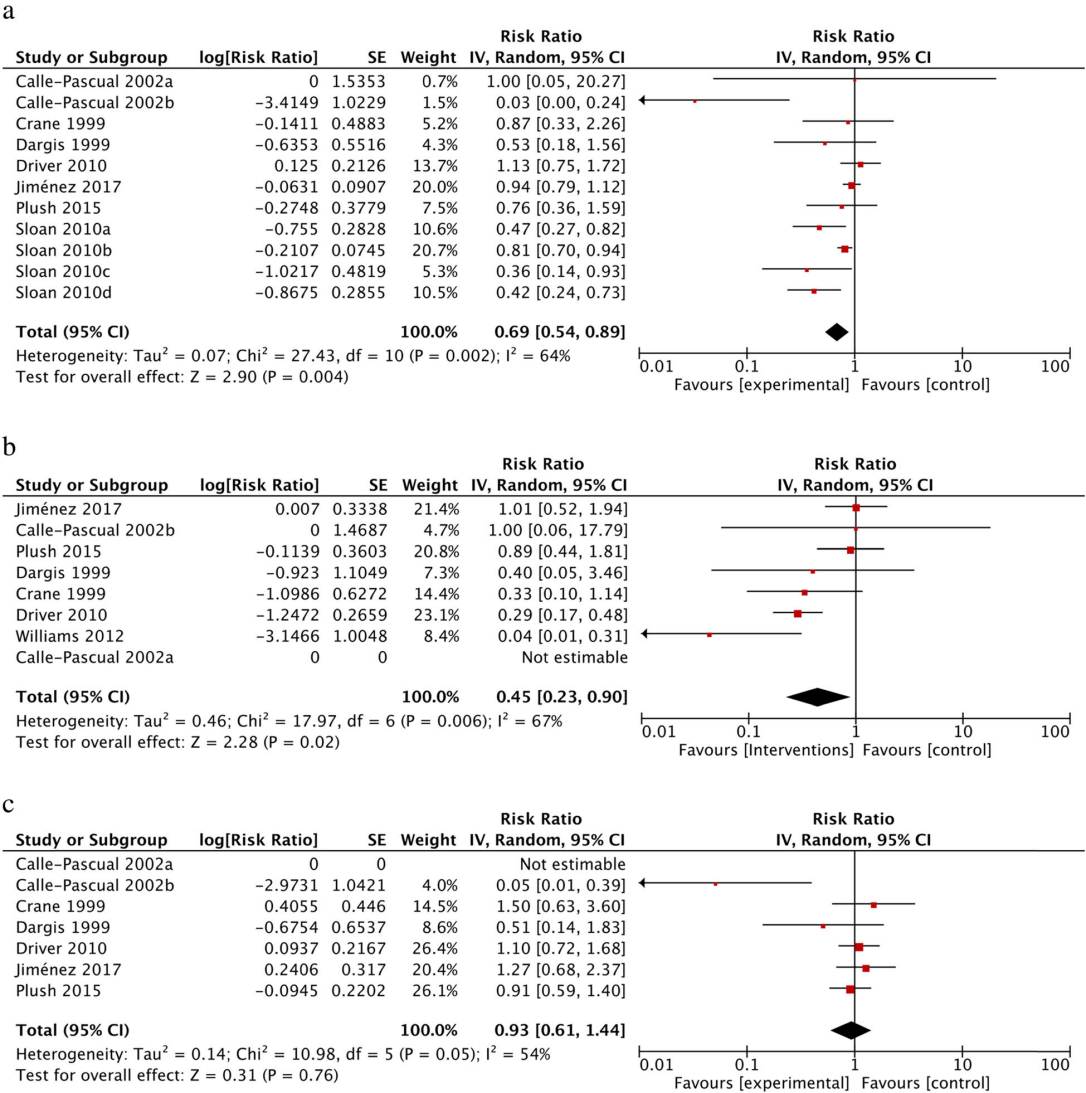
Forest plot for cohort studies **a**) Total LEAs **b**) Major LEAs **c**) Minor LEAs

**Fig. 3:**
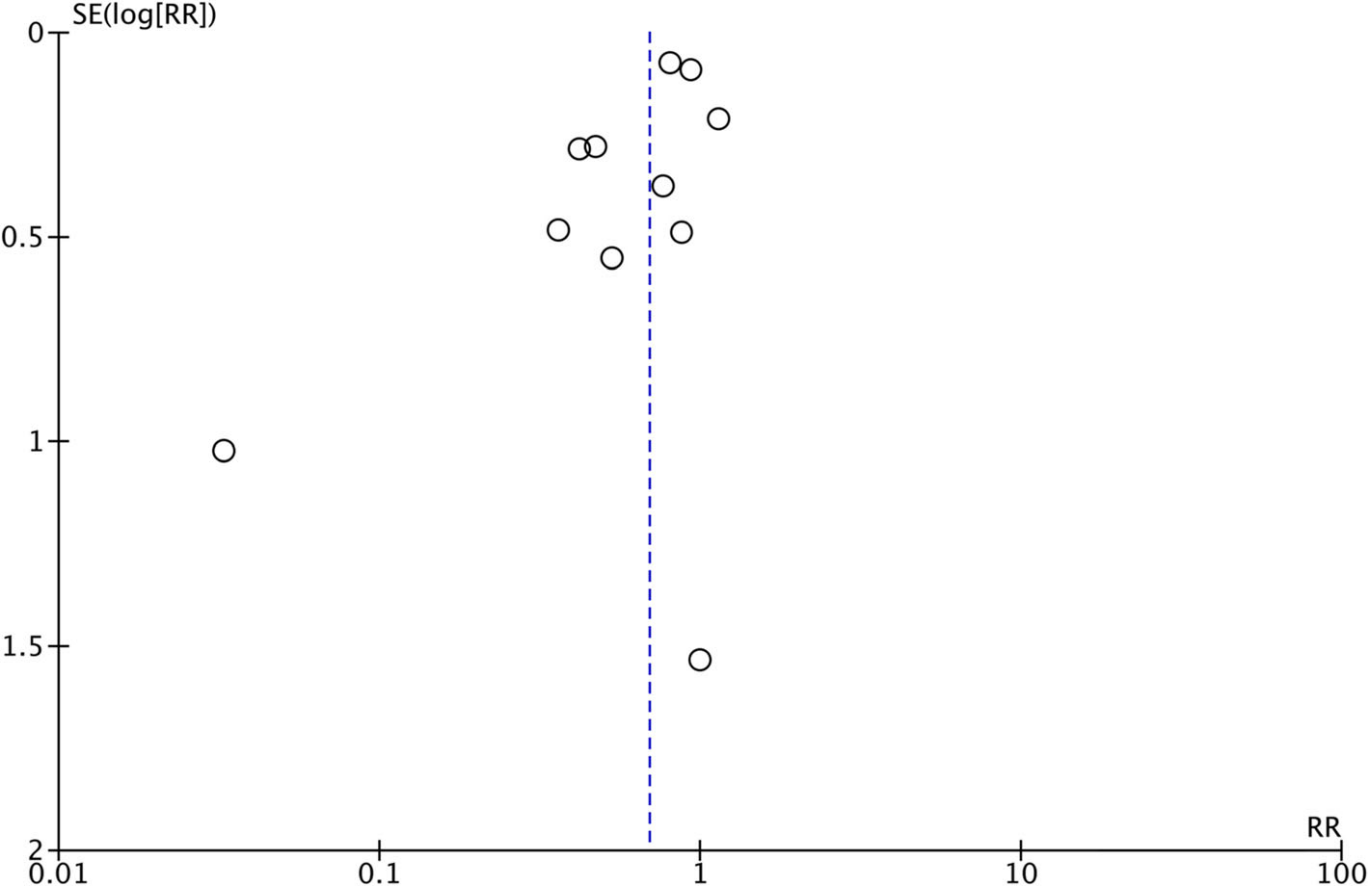
Funnel plot of cohort included studies for total LEAs

All the studies included in the meta-analysis (*n* = 12) reported favourable data for people with diabetes in an MDT management that included podiatry. Therefore, we retrieved data related to our pre-defined outcomes about DFUs and LEAs as stated in Table 1. All included articles had data concerning primary outcomes: LEAs [36, 38, 41, 54–58, 60, 62, 65] and DFUs [38, 54, 55, 57, 58]. With regard to the 11 studies excluded for the meta-analysis, but included in the systematic review (*n* = 23), 10 out of 11 studies reported data in favour of MDTs including podiatry [40, 42, 44–47, 49–51, 53] and one article reported no effect of the interventions [43]. That led us to conduct two separate meta-analyses based on study design (see Fig. 1). Main results are shown in Fig. 2 from available data pooled together, which respects criteria of heterogeneity. For total LEAs as the primary outcome, the random effect model was applied and a significant result was found in favour of MDTs with podiatry (RR: 0.69, 95% CI 0.54–0.89, I^2^ = 64%, *P* = 0.002). For major LEAs (level defined as above knee amputation and/or below knee amputation), results were also in favour of MDTs with podiatry and still significant (RR: 0.45, 95% CI 0.23–0.90, I^2^ = 67%, *P* < 0.02). The result was not significant for minor LEAs (level defined as amputations at any level of the foot) (RR: 0.93, 95% CI 0.59–1.40, I^2^ = 55%, *P* = 0.76). We succeeded in pooling results from 2 pre and post cohorts’ with cohort study analysis, which increased the number of studies included to 8 for meta-analysis. Raw data from these 2 studies allowed us to calculate the prevalence of LEAs per year per period pre-and post-intervention from a sample size based on government census data in the area. Therefore, events of LEAs from exposed group to MDTs and non-exposed group to MDTs were calculated [36, 56]. For the remaining pre and post cohort (*n* = 4) [55, 58–60], because of the significant heterogeneity between studies, we decided not to pool the data with association measure. Pre and post cohort MDTs have reported significant results in favour of MDTs to improve DFU healing rate [55, 58] and reduce total LEA [58, 59] and major LEA [58–60]. Visual inspection of the funnel plot for the included cohort studies for total LEAs has demonstrated no strong evidence of publication bias of the studies in favour of the interventions (Fig. 3). The heterogeneity in DFU data has not allowed meta-analysis for cohort studies.

### Secondary outcomes

According to our predefined secondary outcomes (Table 1), data was available for mortality/survival [36, 41, 43, 54, 58], recurrence [43, 54, 57], other complications [54, 61, 64], and healthcare data [49, 54, 56, 57, 59, 61, 63, 64]. Meta-analyses were performed for some studies, but heterogeneity was over 75%. No articles reported data concerning patients’ satisfaction with care provided by MDTs.

### Risk of bias assessment of included studies

**Fig. 4:**
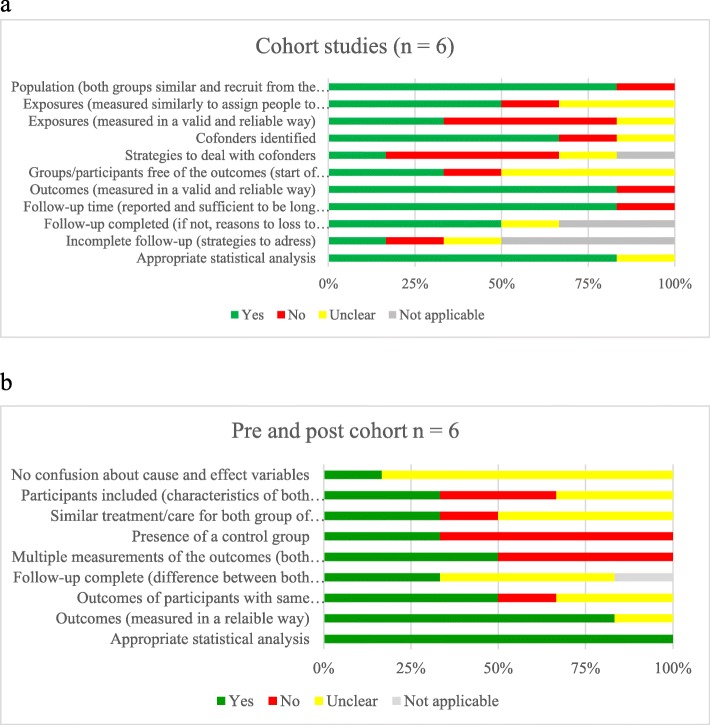
Potential risk of bias with JBI tools **a**) Cohort studies **b**) Pre and post cohort studies

In relation to the critical appraisal of quality and experimental designs, bias analyses for cohorts have shown that none of the included studies fulfilled all parameters for low risk of bias, but the majority of the studies included (4/6) had a low risk of bias for the following parameters: population, confounders identified, outcomes measured, follow-up time, and appropriate statistical analysis. High risk of bias was present concerning the baseline population (those who were not free of LEAs or DFUs at the beginning of the study) and the exposure (valid and reliable method to measure MDTs contact and intervention) (see Fig. 4a). Bias analysis for pre and post cohorts have also shown the same trend of high risk of bias in included studies. None of the included studies fulfilled all parameters for low risk of bias, but the majority of the studies included (4/6) had a low risk of bias for 2 parameters: outcome measurements and appropriate statistical analysis. In almost all studies, there is confusion about the cause and effect variables (5/6) and difference about follow-up time between pre and post cohorts (4/6). Exposition to intervention was a low risk of bias for only 2 study out of 6. Few studies had a control group (2/6) (see Fig. 4b).

## Discussion

A rigorous systematic search of the literature led to the inclusion of 8 studies in a meta-analysis performed to answer our research question. This was allowed because the heterogeneity of included studies, depending on the outcomes, was lower than 75% and the Chi-square test result was less than 30% with significant *p*-value (IC 90%) [33]. The ultimate aim in diabetic foot care is to avoid DFUs and resulting LEAs for individuals with diabetes. The goals and benefits from an MDT that includes a podiatrist reside in complementary work and synergy of skills and knowledge to achieve best outcomes for the patients [65]. This was addressed in all included studies on MDTs that included podiatry. However, even though our study has looked closely at different podiatric interventions in an MDT, there is not enough reported information and descriptions of interventions to examine specific podiatric interventions’ efficiency. Despite this, from the information available, interventions were mainly educational strategies and foot care strategies. It becomes problematic to distinguish precisely whether it is the intervention as performed specifically by a podiatrist that is effective or if it is the intervention itself. According to the interventions described in the included studies, podiatric interventions could have been done by other team members (for example, by a nurse). The evidence of value added by podiatrists in an MDT remained weak in that context. It would have been relevant to have a description of interventions requiring specific podiatrist skills and knowledge in the MDT such as foot surgeries and offloading, which are interventions highly recommended in guidelines for DFU management and very specific to the podiatry competency framework [28]. Only one study integrated specific competencies in their MDT management [47]. For these reasons, we have analysed the relative effect of contact with MDTs that include podiatric interventions as a relative reduction of risk.

### Clinical significance

The results of this systematic review support the concept that MDTs with podiatrists lead to a statistically significant reduction of LEAs (total and major LEAs) compared to interventions without MDTs. After qualitative analysis, authors of the included studies examining minor LEAs as outcomes (all except [38, 61]) have shown that there are more minor LEAs with MDT interventions. However, upon analysis of results in relation with other severities of LEAs (major versus minor) and with total LEAs, level of LEAs may decrease with an MDT with podiatry management. There is a 31% relative risk reduction in undergoing a LEA, either major or minor, with MDT management with podiatry for people at risk for diabetic foot. Considering only major LEAs, the relative risk reduction was of 55%. These results are clinically meaningful in favour of the intervention, considering the high 5-year mortality rate and the low quality of life of patients with diabetes who undergo LEAs [4, 5]. Even if these results are consistent with the current literature, this should be interpreted with caution. Hence, this review cannot make any new recommendations about practice due to several methodological flaws discovered during quality appraisal of the included studies.

### Literature comparison and findings

Three Cochrane reviews of interventions that evaluated the outcomes of LEAs or DFUs in patients with diabetes concluded that there is insufficient evidence that complex interventions and educational interventions can reduce the risk of LEA or DFUs [66–68]. A fourth review, from the International Working Group on the Diabetic Foot, concluded that integrated foot care in MDTs can prevent DFUs in at-risk patients [69]. These authors also mentioned substantial heterogeneity between articles concerning the description of team members, interventions, and design. Previous reviews did not attempt to single out one member in particular, contrary to this systematic review which focuses on podiatry, but have suggested focusing on similarities of team makeups to help determine the real impact [12, 13]. In general, these studies highlighted the complexity of comparing the results of team work from one study to another to draw conclusions, particularly with teams which did not have the exact same set of skills and organization. Research of true effect size with the specific criterion of contact with podiatry, could have helped to assess the collective effort in MDTs. A common conclusion from all of these previous publications was that high-quality evidence from included studies is lacking. Our findings are also coherent with other reviews about the effectiveness of MDTs in reducing major LEA [14, 15].

Studies included in this systematic review were very heterogeneous, as it was concluded in previous systematic reviews. Confounders and risk factors for LEAs and DFUs are well known in people with diabetes [70, 71]. Few studies presented a strategy of risk classification in management (5/12) that allowed us to split the cohort according to the risk [38, 41], but it was not possible for the other articles [54, 59, 60]. Even with the efforts of stratification of the risk for the population at baseline (low risk to active DFUs) across the studies to pool the results, the characteristics of the included populations were sometimes not specified [36, 41, 42, 48]. The baseline population can also lead to poor prognoses, independently of the interventions. Such was the case for patients presenting with infections, gangrene, necrosis, PVD, and Charcot neuroarthropathy at baseline [52, 56, 59, 61, 63, 64, 72]. These intrinsic variations of the population within a study are a factor that explains heterogeneity of the results and the gap with the true effect size.

### Limitations and strengths

To the best of our knowledge, this is the first systematic review that investigates contact with podiatry in an MDT context on the occurrence of LEAs in individuals with diabetes. It was also a first attempt to describe podiatric interventions specifically in MDTs. This had been suggested for further work from a previous meta-analysis [24]. The strengths of this systematic review are the rigorous search strategies, including an attempt to address the risk of bias. The relevance of the findings to clinical practice is coherent with the recommendations of different diabetes associations and organisations which support MDT management of DFUs, such as the American Diabetes Association, the Canadian Diabetes Association, and the International Working Group on the Diabetic Foot, to name only a few examples. Although these recommendations are mainly based on retrospective cohort studies, it highlights the need for research with stronger designs like RCTs to avoid confounding factors and confusion with cause and effect variables. Moreover, the majority of studies have been published in the last decade, which reflects the growing interest for MDTs and interdisciplinary management of DFUs.

This review has limitations that need to be considered when interpreting the results. The available data is largely derived from retrospective cohorts and pre and post cohorts. Therefore, there is a limited ability to determine true association between interventions and outcomes. Observational studies are also not the preferred design for meta-analysis. The review was also limited by unavailable data or data that precluded us from pooling the effect size even after multiple outreaches to authors. One important concern regarding the high risk of bias of the included studies arose mainly due to insufficiency of reporting within the studies, making many criteria unclear. None of the included studies declared whether the researchers had played a role in the delivery of care in the MDT. Because we looked at the specific role of the podiatrist, podiatrist researchers could introduce a bias in favour of the intervention. Another concern is the heterogeneity of populations and confounding factors. Authors also agree that studies included in the meta-analysis are heterogeneous in terms of methodology. This is explained by nonblinded studies with no control groups and the difficulties in addressing biases and confounders in retrospective studies. In addition, we pooled unadjusted association measure data from observational studies. Although we made every attempt to address heterogeneity by conducting subgroup analyses, it made no difference because of the small number of studies included.

There is a need to seek further evidence concerning the effect of interventions for patients with diabetes and to determine the role of podiatrists in MDTs related to guideline recommendations [16, 19, 24, 73]. Moreover, more studies with stronger designs and methods are needed to determine the effects of interventions on DFUs and LEAs, with a special concern about risk stratification in their population to avoid confounding factors.. The research process should begin with better published studies. Future MDTs could also benefit from addressing timing and care trajectories with stronger descriptions of their specific interventions. It would also be interesting to look at the impact between different populations (low risk versus high risk) and of other team members’ interventions.

## Conclusion

This systematic review of interventions concerning outcomes of LEAs in individuals with diabetes concludes that there is insufficient evidence that MDTs with podiatry management can reduce the risk of LEAs. Even with a favourable outcome of the intervention, the lack of high-quality studies included and considerable heterogeneity nuanced the results concerning relative risk reduction for total and major LEAs. This systematic review’s conclusion follows the same direction as previous literature concerning the management of at-risk diabetic foot in MDTs, and supports previous conclusions about the problem of heterogeneity concerning MDT specialists that manage at-risk diabetic foot and the lack of intervention description (nature, intensity, duration, time of exposure, etc.).

## Supplementary information

**Additional file 1.** Search strategy for Medline via EBSCO (1971).

**Additional file 2.** JBI Critical Appraisal Checklist for Cohort Studies [68].

**Additional file 3.** Articles from reference searching.

**Additional file 4.** Study (author, country, year), Exclusion criteria, Details.

**Additional file 5.** Characteristics of included studies for meta-analysis – Contact with podiatrist in MDT context.
