# Supplementary material for: Effect of contact with podiatry in a team approach context on diabetic foot ulcer and lower extremity amputation: systematic review and meta-analysis
Source: J Foot Ankle Res. 2020 Mar 20;13:15. doi: 10.1186/s13047-020-0380-8 (PMC7083052; doi:10.1186/s13047-020-0380-8)
Supplement: Supplementary file 1 — Additional file 1. Search strategy for Medline via EBSCO (1971). [file 13047_2020_380_MOESM1_ESM.docx]

# Appendix A

### Search strategy for Medline via EBSCO (1971 to February 1^st^, 2020)

1. (MH "Diabetes Mellitus+") OR (MH "Diabetes Mellitus, Type 2+") OR (MH "Diabetes Mellitus, Type 1+") OR "diabetes"
2. diabet*
3. 1 OR 2
4. (MM "Podiatry") OR "podiatry"
5. podiatr* OR chiropod* OR podolog*
6. 4 OR 5
7. (MH "Amputation+") OR "amputation"
8. amput*
9. (MM "Diabetic Foot") OR (MM "Foot Ulcer+") OR (MM "Ulcer") OR "diabetic foot ulceration"
10. foot ulcer* OR mal perforans
11. 7 OR 8 OR 9 OR 10 OR
12. (MH "Cohort Studies+") OR "cohort studies" OR (MM "Retrospective Studies") OR (MM "Prospective Studies")
13. TX cohort*
14. (MH "Case-Control Studies+") OR (MH "Controlled Before-After Studies") OR "case control studies"
15. TX case-control*
16. (MH "Clinical Trials as Topic+") OR (MH "Controlled Clinical Trials as Topic+") OR (MH "Non-Randomised Controlled Trials as Topic") OR (MH "Randomized Controlled Trials as Topic+") OR "clinical trials"
17. Non-Randomized Controlled Trial*
18. Randomised Controlled Trial*
19. 12 OR 13 OR 14 OR 15 OR 16 OR 17 OR 18
20. 3 AND 6 AND 11 AND 19
21. (MH "Patient Care Team+") OR "patient care team" OR (MH "Patient-Centered Care+") OR (MH "Patient Care+")
22. multidisciplinar*
23. interdisciplinar*
24. team approach*
25. team work OR teamwork OR team work*
26. 21 OR 22 OR 23 OR 24 OR 25
27. 20 AND 26

**Total : 1 557**

### Search strategy for CINAHL via EBSCO (1981 to February 1^st^, 2020)

1. (MH "Diabetes Mellitus+") OR (MH "Diabetes Mellitus, Type 2+") OR (MH "Diabetes Mellitus, Type 1+") OR "diabetes"
2. diabet*
3. 1 OR 2
4. (MM "Podiatry") OR "podiatry"
5. podiatr* OR chiropod* OR podolog*
6. 4 OR 5
7. (MH "Amputation+") OR "amputation" OR "limb loss"
8. amput*
9. (MM "Diabetic Foot") OR (MM "Foot Ulcer+") OR (MM "Ulcer") OR "diabetic foot ulceration"
10. foot ulcer* OR mal perforan*
11. 7 OR 8 OR 9 OR 10 OR
12. (MH "Cohort Studies+") OR "cohort studies" OR (MM "Retrospective Studies") OR (MM "Prospective Studies")
13. TX cohort*
14. (MH "Case-Control Studies+") OR (MH "Controlled Before-After Studies") OR "case control studies"
15. TX case-control*
16. (MH "Clinical Trials as Topic+") OR (MH "Controlled Clinical Trials as Topic+") OR (MH "Non-Randomised Controlled Trials as Topic") OR (MH "Randomized Controlled Trials as Topic+") OR "clinical trials"
17. Non-Randomised Controlled Trial*
18. Randomised Controlled Trial*
19. 12 OR 13 OR 14 OR 15 OR 16 OR 17 OR 18
20. 3 AND 6 AND 11 AND 19
21. (MH "Patient Care Team+") OR "patient care team" OR (MH "Patient-Centered Care+") OR (MH "Patient Care+")
22. multidisciplinar*
23. interdisciplinar*
24. team approach*
25. team work OR teamwork OR team work*
26. collaboration*
27. 21 OR 22 OR 23 OR 24 OR 25 OR 26
28. 20 AND 27

**Total : 1583**

### Search strategy for Cochrane (1993 to February 1^st^, 2020)

1. MeSH descriptor [Diabetes Mellitus] explode all trees
2. diabet*
3. 1 or 2
4. MeSH descriptor [Podiatry] explode all trees
5. podiatr*
6. chiropod*
7. podolog*
8. MeSH descriptor: [Patient Care Team] explode all trees
9. multidisciplinar*
10. interdisciplinar*
11. team approach
12. team work* or teamwork*
13. 4 or 5 or 6 or 7 or 8 or 9 or 10 or 11 or 12
14. MeSH descriptor [Foot Ulcer] explode all trees
15. foot ulcer*
16. mal perforan*
17. diabetic wound*
18. MeSH descriptor [Amputation] explode all trees
19. amput*
20. 14 or 15 or 16 or 17 or 18 or 19
21. 20 and 13 and 3

**Total : 423**

242 Cochrane Reviews

1 Cochrane protocole

6 Other Reviews

1 Method Studies

161 Trials

6 Technology Assessments

11 Economic Evaluation

6 Cochrane Groups

### Search strategy for EMBASE via Ovid (1974 to February 1^st^, 2020)

1. exp diabetes mellitus/
2. diabetes mellitus.mp.
3. diabet*.mp.
4. 1 or 2 or 3
5. exp podiatry/
6. podiatry/ or podiatry.mp.
7. podiatr*.mp.
8. chiropod*.mp.
9. podolog*.mp.
10. 5 or 6 or 7 or 8 or 9
11. foot amputation/ or knee amputation/ or amputation/ or below knee amputation/ or above knee amputation/ or leg amputation/ or limb amputation/
12. amputation.mp.
13. amput*.mp.
14. 11 or 12 or 13
15. diabetic neuropathy/ or diabetic foot/ or ulcer/ or foot ulcer/
16. plantar ulcer/ or leg ulcer/ or ulcer/ or ulcer*.mp. or ulcer perforation/ or application site ulcer/ or ulcer healing/ or foot ulcer/ or skin ulcer/
17. mal perforans.mp.
18. foot ulcer*.mp.
19. 15 or 16 or 17 or 18
20. 14 or 19
21. patient care team.mp. or exp patient care/
22. multidisciplinary.mp.
23. team approach.mp. or exp teamwork/
24. interdisciplinar*.mp.
25. 21 or 22 or 23 or 24
26. 10 or 25
27. case control studies.mp. or case control study/
28. cohort study.mp. or cohort analysis/
29. retrospective study.mp. or retrospective study/
30. prospective study.mp. or prospective study/
31. clinical trial/
32. randomised clinical trial*.mp. or non-randomized clinical trial*.mp
33. 27 or 28 or 29 or 30 or 31 or 32
34. 4 and 20 and 26 and 33

**Total : 1 424**
