# Supplementary material for: Effect of contact with podiatry in a team approach context on diabetic foot ulcer and lower extremity amputation: systematic review and meta-analysis
Source: J Foot Ankle Res. 2020 Mar 20;13:15. doi: 10.1186/s13047-020-0380-8 (PMC7083052; doi:10.1186/s13047-020-0380-8)
Supplement: Supplementary file 2 — Additional file 2. JBI Critical Appraisal Checklist for Cohort Studies [68]. [file 13047_2020_380_MOESM2_ESM.docx]

## –––Appendix B

**JBI Critical Appraisal Checklist for Cohort Studies [68]**

|  | Yes | No | Unclear | Not applicable |
| --- | --- | --- | --- | --- |
| 1. Were the two groups similar and recruited from the same population? | □ | □ | □ | □ |
| 1. Were the exposures measured similarly to assign people   to both exposed and unexposed groups? | □ | □ | □ | □ |
| 1. Was the exposure measured in a valid and reliable way? | □ | □ | □ | □ |
| 1. Were confounding factors identified? | □ | □ | □ | □ |
| 1. Were strategies to deal with confounding factors stated? | □ | □ | □ | □ |
| 1. Were the groups/participants free of the outcome at the start of the study (or at the moment of exposure)? | □ | □ | □ | □ |
| 1. Were the outcomes measured in a valid and reliable way? | □ | □ | □ | □ |
| 1. Was the follow up time reported and sufficient to be long enough for outcomes to occur? | □ | □ | □ | □ |
| 1. Was follow up complete, and if not, were the reasons to loss to follow up described and explored? | □ | □ | □ | □ |
| 1. Were strategies to address incomplete follow up utilized? | □ | □ | □ | □ |
| 1. Was appropriate statistical analysis used? | □ | □ | □ | □ |

Overall appraisal: Include □ Exclude □ Seek further info □

Comments (Including reason for exclusion)

**Explanations of criteria**

1. **Were the two groups similar and recruited from the same population?**

Check the paper carefully for descriptions of participants to determine if patients within and across groups have similar characteristics in relation to exposure (e.g. risk factor under investigation). The two groups selected for comparison should be as similar as possible in all characteristics except for their exposure status, relevant to the study in question. The authors should provide clear inclusion and exclusion criteria that they developed prior to recruitment of the study participants.

1. **Were the exposures measured similarly to assign people to both exposed and unexposed groups?**

A high quality study at the level of cohort design should mention or describe how the exposures were measured. The exposure measures should be clearly defined and described in detail. This will enable reviewers to assess whether or not the participants received the exposure of interest.

1. **Was the exposure measured in a valid and reliable way?**

The study should clearly describe the method of measurement of exposure. Assessing validity requires that a 'gold standard' is available to which the measure can be compared. The validity of exposure measurement usually relates to whether a current measure is appropriate or whether a measure of past exposure is needed.

Reliability refers to the processes included in an epidemiological study to check repeatability of measurements of the exposures. These usually include intra-observer reliability and inter-observer reliability.

1. **Were confounding factors identified?**

Confounding has occurred where the estimated intervention exposure effect is biased by the presence of some difference between the comparison groups (apart from the exposure investigated/of interest). Typical confounders include baseline characteristics, prognostic factors, or concomitant exposures (e.g. smoking). A confounder is a difference between the comparison groups and it influences the direction of the study results. A high quality study at the level of cohort design will identify the potential confounders and measure them (where possible). This is difficult for studies where behavioral, attitudinal or lifestyle factors may impact on the results.

1. **Were strategies to deal with confounding factors stated?**

Strategies to deal with effects of confounding factors may be dealt within the study design or in data analysis. By matching or stratifying sampling of participants, effects of confounding factors can be adjusted for. When dealing with adjustment in data analysis, assess the statistics used in the study. Most will be some form of multivariate regression analysis to account for the confounding factors measured. Look out for a description of statistical methods as regression methods such as logistic regression are usually employed to deal with confounding factors/variables of interest.

1. **Were the groups/participants free of the outcome at the start of the study (or at the moment of exposure)?**

The participants should be free of the outcomes of interest at the start of the study. Refer to the ‘methods’ section in the paper for this information, which is usually found in descriptions of participant/sample recruitment, definitions of variables, and/or inclusion/exclusion criteria.

1. **Were the outcomes measured in a valid and reliable way?**

Read the methods section of the paper. If for e.g. lung cancer is assessed based on existing definitions or diagnostic criteria, then the answer to this question is likely to be yes. If lung cancer is assessed using observer reported, or self-reported scales, the risk of over- or under-reporting is increased, and objectivity is compromised. Importantly, determine if the measurement tools used were validated instruments as this has a significant impact on outcome assessment validity.

Having established the objectivity of the outcome measurement (e.g. lung cancer) instrument, it’s important to establish how the measurement was conducted. Were those involved in collecting data trained or educated in the use of the instrument/s? (e.g. radiographers). If there was more than one data collector, were they similar in terms of level of education, clinical or research experience, or level of responsibility in the piece of research being appraised?

1. **Was the follow up time reported and sufficient to be long enough for outcomes to occur?**

The appropriate length of time for follow up will vary with the nature and characteristics of the population of interest and/or the intervention, disease or exposure. To estimate an appropriate duration of follow up, read across multiple papers and take note of the range for duration of follow up. The opinions of experts in clinical practice or clinical research may also assist in determining an appropriate duration of follow up. For example, a longer timeframe may be needed to examine the association between occupational exposure to asbestos and the risk of lung cancer. It is important, particularly in cohort studies that follow up is long enough to enable the outcomes. However, it should be remembered that the research question and outcomes being examined would probably dictate the follow up time.

1. **Was follow up complete, and if not, were the reasons to loss to follow up described and explored?**

It is important in a cohort study that a greater percentage of people are followed up. As a general guideline, at least 80% of patients should be followed up. Generally a dropout rate of 5% or less is considered insignificant. A rate of 20% or greater is considered to significantly impact on the validity of the study. However, in observational studies conducted over a lengthy period of time a higher dropout rate is to be expected. A decision on whether to include or exclude a study because of a high dropout rate is a matter of judgement based on the reasons why people dropped out, and whether dropout rates were comparable in the exposed and unexposed groups.

Reporting of efforts to follow up participants that dropped out may be regarded as an indicator of a well conducted study. Look for clear and justifiable description of why people were left out, excluded, dropped out etc. If there is no clear description or a statement in this regards, this will be a 'No'.

**10. Were strategies to address incomplete follow up utilized?**

Some people may withdraw due to change in employment or some may die; however, it is important that their outcomes are assessed. Selection bias may occur as a result of incomplete follow up. Therefore, participants with unequal follow up periods must be taken into account in the analysis, which should be adjusted to allow for differences in length of follow up periods. This is usually done by calculating rates which use person-years at risk, i.e. considering time in the denominator.

**11. Was appropriate statistical analysis used?**

As with any consideration of statistical analysis, consideration should be given to whether there was a more appropriate alternate statistical method that could have been used. The methods section of cohort studies should be detailed enough for reviewers to identify which analytical techniques were used (in particular, regression or stratification) and how specific confounders were measured.

For studies utilizing regression analysis, it is useful to identify if the study identified which variables were included and how they related to the outcome. If stratification was the analytical approach used, were the strata of analysis defined by the specified variables? Additionally, it is also important to assess the appropriateness of the analytical strategy in terms of the assumptions associated with the approach as differing methods of analysis are based on differing assumptions about the data and how it will respond.

**JBI Critical Appraisal Checklist for Quasi-experimental studies [68]**

|  | Yes | No | Unclear | Not applicable |
| --- | --- | --- | --- | --- |
| 1. Is it clear in the study what is the ‘cause’ and what is the ‘effect’ (i.e. there is no confusion about which variable comes first)? | □ | □ | □ | □ |
| 1. Were the participants included in any comparisons similar? | □ | □ | □ | □ |
| 1. Were the participants included in any comparisons receiving similar treatment/care, other than the exposure or intervention of interest? | □ | □ | □ | □ |
| 1. Was there a control group? | □ | □ | □ | □ |
| 1. Were there multiple measurements of the outcome both pre and post the intervention/exposure? | □ | □ | □ | □ |
| 1. Was follow up complete and if not, were differences between groups in terms of their follow up adequately described and analyzed? | □ | □ | □ | □ |
| 1. Were the outcomes of participants included in any comparisons measured in the same way? | □ | □ | □ | □ |
| 1. Were outcomes measured in a reliable way? | □ | □ | □ | □ |
| 1. Was appropriate statistical analysis used? | □ | □ | □ | □ |

Overall appraisal: Include □ Exclude □ Seek further info □

Comments (Including reason for exclusion)

**Explanation for the criteria**

1. **Is it clear in the study what is the ‘cause’ and what is the ‘effect’ (i.e. there is no confusion about which variable comes first)?**

Ambiguity with regards to the temporal relationship of variables constitutes a threat to the internal validity of a study exploring causal relationships. The ‘cause’ (the independent variable, that is, the treatment or intervention of interest) should occur in time before the explored ‘effect’ (the dependent variable, which is the effect or outcome of interest). Check if it is clear which variable is manipulated as a potential cause. Check if it is clear which variable is measured as the effect of the potential cause. Is it clear that the ‘cause’ was manipulated before the occurrence of the ‘effect’?

**2. Were the participants included in any comparisons similar?**

The differences between participants included in compared groups constitute a threat to the internal validity of a study exploring causal relationships. If there are differences between participants included in compared groups there is a risk of selection bias. If there are differences between participants included in the compared groups maybe the ‘effect’ cannot be attributed to the potential ‘cause’, as maybe it is plausible that the ‘effect’ may be explained by the differences between participants, that is, by selection bias. Check the characteristics reported for participants. Are the participants from the compared groups similar with regards to the characteristics that may explain the effect even in the absence of the ‘cause’, for example, age, severity of the disease, stage of the disease, co-existing conditions and so on? *[NOTE: In one single group pre-test/post-test studies where the patients are the same (the same one group) in any pre-post comparisons, the answer to this question should be ‘yes.’]*

**3. Were the participants included in any comparisons receiving similar treatment/care, other than the exposure or intervention of interest?**

In order to attribute the ‘effect’ to the ‘cause’ (the exposure or intervention of interest), assuming that there is no selection bias, there should be no other difference between the groups in terms of treatments or care received, other than the manipulated ‘cause’ (the intervention of interest). If there are other exposures or treatments occurring in the same time with the ‘cause’, other than the intervention of interest, then potentially the ‘effect’ cannot be attributed to the intervention of interest, as it is plausible that the ‘effect’ may be explained by other exposures or treatments, other than the intervention of interest, occurring in the same time with the intervention of interest. Check the reported exposures or interventions received by the compared groups. Are there other exposures or treatments occurring in the same time with the intervention of interest? Is it plausible that the ‘effect’ may be explained by other exposures or treatments occurring in the same time with the intervention of interest?

4. **Was there a control group?**

Control groups offer the conditions to explore what would have happened with groups exposed to other different treatments, other than to the potential ‘cause’ (the intervention of interest). The comparison of the treated group (the group exposed to the examined ‘cause’, that is, the group receiving the intervention of interest) with such other groups strengthens the examination of the causal plausibility. The validity of causal inferences is strengthened in studies with at least one independent control group compared to studies without an independent control group. Check if there are independent, separate groups, used as control groups in the study. *[Note: The control group should be an independent, separate control group, not the pre-test group in a single group pre-test post-test design.]*

**5. Were there multiple measurements of the outcome both pre and post the intervention/exposure?**

In order to show that there is a change in the outcome (the ‘effect’) as a result of the intervention/treatment (the ‘cause’) it is necessary to compare the results of measurement before and after the intervention/treatment. If there is no measurement before the treatment and only measurement after the treatment is available it is not known if there is a change after the treatment compared to before the treatment. If multiple measurements are collected before the intervention/treatment is implemented then it is possible to explore the plausibility of alternative explanations other than the proposed ‘cause’ (the intervention of interest) for the observed ‘effect’, such as the naturally occurring changes in the absence of the ‘cause’, and changes of high (or low) scores towards less extreme values even in the absence of the ‘cause’ (sometimes called regression to the mean). If multiple measurements are collected after the intervention/treatment is implemented it is possible to explore the changes of the ‘effect’ in time in each group and to compare these changes across the groups. Check if measurements were collected before the intervention of interest was implemented. Were there multiple pre-test measurements? Check if measurements were collected after the intervention of interest was implemented. Were there multiple post-test measurements?

**6. Was follow up complete and if not, were differences between groups in terms of their follow up adequately described and analyzed?**

If there are differences with regards to the loss to follow up between the compared groups these differences represent a threat to the internal validity of a study exploring causal effects as these differences may provide a plausible alternative explanation for the observed ‘effect’ even in the absence of the ‘cause’ (the treatment or exposure of interest). Check if there were differences with regards to the loss to follow up between the compared groups. If follow up was incomplete (that is, there is incomplete information on all participants), examine the reported details about the strategies used in order to address incomplete follow up, such as descriptions of loss to follow up (absolute numbers; proportions; reasons for loss to follow up; patterns of loss to follow up) and impact analyses (the analyses of the impact of loss to follow up on results). Was there a description of the incomplete follow up (number of participants and the specific reasons for loss to follow up)? If there are differences between groups with regards to the loss to follow up, was there an analysis of patterns of loss to follow up? If there are differences between the groups with regards to the loss to follow up, was there an analysis of the impact of the loss to follow up on the results?

**7. Were the outcomes of participants included in any comparisons measured in the same way?**

If the outcome (the ‘effect’) is not measured in the same way in the compared groups there is a threat to the internal validity of a study exploring a causal relationship as the differences in outcome measurements may be confused with an effect of the treatment or intervention of interest (the ‘cause’). Check if the outcomes were measured in the same way. Same instrument or scale used? Same measurement timing? Same measurement procedures and instructions?

**8. Were outcomes measured in a reliable way?**

Unreliability of outcome measurements is one threat that weakens the validity of inferences about the statistical relationship between the ‘cause’ and the ‘effect’ estimated in a study exploring causal effects. Unreliability of outcome measurements is one of different plausible explanations for errors of statistical inference with regards to the existence and the magnitude of the effect determined by the treatment (‘cause’). Check the details about the reliability of measurement such as the number of raters, training of raters, the intra-rater reliability, and the inter-raters reliability within the study (not to external sources). This question is about the reliability of the measurement performed in the study, it is not about the validity of the measurement instruments/scales used in the study. *[Note: Two other important threats that weaken the validity of inferences about the statistical relationship between the ‘cause’ and the ‘effect’ are low statistical power and the violation of the assumptions of statistical tests. These other threats are not explored within Question 8, these are explored within Question 9.]*

**9. Was appropriate statistical analysis used?**

Inappropriate statistical analysis may cause errors of statistical inference with regards to the existence and the magnitude of the effect determined by the treatment (‘cause’). Low statistical power and the violation of the assumptions of statistical tests are two important threats that weakens the validity of inferences about the statistical relationship between the ‘cause’ and the ‘effect’. Check the following aspects: if the assumptions of statistical tests were respected; if appropriate statistical power analysis was performed; if appropriate effect sizes were used; if appropriate statistical procedures or methods were used given the number and type of dependent and independent variables, the number of study groups, the nature of the relationship between the groups (independent or dependent groups), and the objectives of statistical analysis (association between variables; prediction; survival analysis etc.).
