# Supplementary material for: Effect of contact with podiatry in a team approach context on diabetic foot ulcer and lower extremity amputation: systematic review and meta-analysis
Source: J Foot Ankle Res. 2020 Mar 20;13:15. doi: 10.1186/s13047-020-0380-8 (PMC7083052; doi:10.1186/s13047-020-0380-8)
Supplement: Supplementary file 3 — Additional file 3. Articles from reference searching. [file 13047_2020_380_MOESM3_ESM.docx]

**Appendix C**

### Articles from reference searching

1. Eskelinen, E., et al. "Major amputation incidence decreases both in non-diabetic and in diabetic patients in Helsinki." Scandinavian Journal of Surgery 95.3 (2006): 185-189.
2. Martin, Toni L., Joe V. Selby, and Danya Zhang. "Physician and patient prevention practices in NIDDM in a large urban managed-care organization." Diabetes Care 18.8 (1995): 1124-1132..
3. Bild, Diane E., et al. "Lower-extremity amputation in people with diabetes: epidemiology and prevention." Diabetes care 12.1 (1989): 24-31.
4. Burden, A. C., A. Samanta, and Rosemary Jones. "Setting up an advanced foot clinic in a District General Hospital." Practical Diabetes International 3.5 (1986): 262-262.Baillie 2017
5. Thomson, F. J., et al. "A team approach to diabetic foot care—the Manchester experience." *The Foot* 1.2 (1991): 75-82
6. Baillie, C., et al. "Multidisciplinary Approach to the Management of Diabetic Foot Complications: Impact on Hospital Admissions, Limb Salvage and Amputation Rates." Endocrinol Metab Int J 5.2 (2017): 00119.
7. Smith, David M., Morris Weinberger, and Barry P. Katz. "A controlled trial to increase office visits and reduce hospitalizations of diabetic patients." Journal of General Internal Medicine 2.4 (1987): 232-238
8. Keyser, John E. "Diabetic wound healing and limb salvage in an outpatient wound care program." Southern medical journal 86.3 (1993): 311-317.

### Articles from grey literature

1. McCardle, J., Chadwick, P., Leese, G., Mcinnes, Alistair, Stang, D., Stuart, L. and Young, M. (2012) Podiatry competency framework for integrated diabetic foot care: a user's guide SB Communications Group, London, UK
2. International woking group on the Diabetic foot guidelines [Accessible online] <http://iwgdf.org/guidelines/>) or Bakker, K., N. C. Schaper, and International Working Group on the Diabetic Foot Editorial Board. "The development of global consensus guidelines on the management and prevention of the diabetic foot 2011." Diabetes/metabolism research and reviews 28 (2012): 116-118.
