# Supplementary material for: Effect of contact with podiatry in a team approach context on diabetic foot ulcer and lower extremity amputation: systematic review and meta-analysis
Source: J Foot Ankle Res. 2020 Mar 20;13:15. doi: 10.1186/s13047-020-0380-8 (PMC7083052; doi:10.1186/s13047-020-0380-8)
Supplement: Supplementary file 4 — Additional file 4. Study (author, country, year), Exclusion criteria, Details. [file 13047_2020_380_MOESM4_ESM.docx]

**Appendix D**

| **Study (author, country, year)** | **Exclusion criteria** | **Details** |
| --- | --- | --- |
| 1. Bakker and al., Netherlands, 1997 | Design | Cross-sectional study |
| 1. McCabe and al., UK, 1998 | Intervention/Design | Only a part of population had seen podiatrist (interventions) and no randomization |
| 1. Alvarsson and al., Sweden, 2012 | Intervention | Only 30% of population in MDT |
| 1. Sanders and al., Netherlands, 2013 | Intervention | No difference in exposition of intervention between group |
| 1. Rasmussen and al., Denmark, 2017 | Reported elsewhere | Rasmussen and al.,2015 and no control/comparison group |
| 1. Almdal and al., Denmardk, 2015 | Design | No control/comparison group |
| 1. Apelqvist and al., Sweden, 1993 | Design | No control/comparison group |
| 1. Armstrong and al., USA, 1998 | Design | No control/comparison group |
| 1. Bateman and al., UK, 2015 | Design | Cohort review with no control/comparison group |
| 1. Beany and al., UK, 2016 | Design | No control/comparison group |
| 1. Canavan and al., UK, 2008 | Design | No control/comparison group |
| 1. Casey and al., UK, 2018 | Article type | Conference abstract |
| 1. Del Aguila and al., USA, 1994 | Intervention | Exposition to podiatry unclear |
| 1. Driver and al., USA, 2005 | Design | No control/comparison group |
| 1. El Sakka and al., UK, 2006 | Design | No control/comparison group |
| 1. Falkenberg, Seden, 1990 | Design | Cross-sectional study/comparison group |
| 1. Foster and al., UK, 1995 | Design | No control/comparison group |
| 1. Frykberg and al., USA, 1996 | Intervention/Design | Difference between podiatrist exposition, cross sectional study |
| 1. Gershater and al., Sweden, 2008 | Design | No control/comparison group |
| 1. Hamonet and al., France, 2010 | Intervention | Exposition to podiatry unclear and no control/comparison group |
| 1. Hartemann-Heurtier and al., France, 2002 | Design | No control/comparison group |
| 1. Hellingman and al., Netherlands, 2008 | Article type | Audit |
| 1. Hicks and al., USA, 2017 | Intervention | Difference between intervention/podiatrist exposition and no control/comparison group |
| 1. Holstein and al., Denmark, 2000 | Design | Cross-sectional study |
| 1. Jeffcoate and al., UK, 2006 | Article type | Audit |
| 1. Labovitz and al., USA, 2016 | Design | Cross-sectional study |
| 1. Krishnan and al., UK, 2008 | Article type | Audit |
| 1. Kröger and al., Germany, 2014 | Design/Intervention | Difference between intervention/podiatrist exposition, no control/comparison group, cross-sectional study |
| 1. Larsson and al., Sweden, 2008 | Design | Cross-sectional study |
| 1. Larsson and al., Sweden, 1999 | Design | No control/comparison group |
| 1. Leese and al., UK, 2012 | Design | No control/comparison group |
| 1. Lu and al., Canada, 2017 | Design | Cross-sectional study |
| 1. Metcalf and al., UK, 2017 | Design | Cross-sectional study |
| 1. Monteiro-Soares and al., Portugal, 2012 | Reported elsewhere | Monteiro-Soares and al., Portugal, 2010 |
| 1. Monteiro-Soares and al., Portugal, 2010 | Design | Cross-sectional study |
| 1. Maury and al., France, 2015 | Intervention | Difference between intervention/podiatrist exposition and no control/comparison group |
| 1. Nason and al., Ireland, 2010 | Article type | Conference abstract |
| 1. Nielson and al., Denmark, 2012 | Article type | Abstract, also Reported elsewhere Almdal and al., Denmardk, 2015 and Almdal and al., Denmardk, 2015 |
| 1. Mathioudakis and al., USA, 2017 | Intervention/Design | Difference between intervention/podiatrist exposition and no control/comparison group, cross sectional study |
| 1. Ogrin and al., Canada, 2013 | Design | No control/comparison group |
| 1. Örneholm and al., Sweden, 2016 | Design | No control/comparison group |
| 1. O’Rourke and al., Australia, 2012 | Article type | Audit |
| 1. Örneholm and al., Sweden, 2015 | Intervention | Difference between intervention/podiatrist exposition |
| 1. Paisey and al., UK, 2017 | Design | Cross-sectional study |
| 1. Perrin and al., Australia, 2012 | Article type | Audit |
| 1. Pütter and al., Germany, 2016 | Design | Cross-sectional study |
| 1. Rajendran and al., UK, 2012 | Design | Cross-sectional study |
| 1. Robbins and al., UAS, 2006 | Design | Cross-sectional study |
| 1. Rhou and al., Australia, 2015 | Outcomes/ Intervention | Not appropriate outcomes, difference between intervention/podiatrist exposition |
| 1. Steed and al., USA, 1993 | Design | Cross-sectional study |
| 1. Snyder and al., USA, 2006 | Design | No control/comparison group |
| 1. De Sonnaville and al., Netherlands, 1997 | Design | Cross-sectional study |
| 1. Thomas and al., UK, 2010 | Design | No control/comparison group |
| 1. Valdès and al., Norwegian, 1999 | Design | Cross-sectional study |
| 1. Todd and al., USA, 1996 | Design | Cross-sectional study |
| 1. Van Gils and al., USA, 1999 | Design | No control/comparison group |
| 1. Van Acker and al., Belgium, 1999 | Design | Cross-sectional study |
| 1. Zayed and al., UK, 2008 | Design | Cross-sectional study |
| 1. Williams and al., UK, 2017 | Design | No control/comparison group |
| 1. Weck and al., Germany, 2013 | Intervention | Exposition to podiatry unclear |
| 1. Casey and al., UK, 2018 | Article type | Conference abstract |
| 1. Ahmad and al., UK, 2018 | Design | Cross-sectional study |
| 1. Abbott and al., | Intervention | No podiatry association or MDT |
| 1. Van Gils and al, USA, 1999 | Design | No control/comparison group |
| 1. Malone and al., USA, 1989 | Intervention | Education by podiatrists, not as a treatment |
| 1. Ellis and al., New Zealand, 2010 | Article type | Audit |
| 1. Schraer and al., USA2004 | Intervention | Initially podiatrist then, physical therapist as a pedorthist, program |
| 1. Abbott and al., UK, 2002 | Intervention | Podiatry attendance measured in the cohort, not as an intervention |
| 1. Alexandrescu and al., Belgium, 2009 | Design | Case series |
| 1. Buckley and al., Ireland, 2013 | Intervention | Podiatry involved in services studied but not as an intervention |
| 1. Buckley and al., Ireland, 2013 | Design | Systematic review |
| 1. Ali and al., UK, 2014 | Article type | Conference abstract |
| 1. Al and al., UK 2013 | Article type | Conference abstract |
| 1. Ali and al., Pakistan, 2008 | Intervention | Information retrieved from chiropody service but not as intervention |
| 1. Anichini and al., Italy, 2012 | Article type | Conference abstract, unpublished data |
| 1. Apelqvist and al., Sweden, 1999 | Language | Article written in Swedish |
| 1. Apelqvist and al., Sweden, 1994 | Design | Case series / no comparison |
| 1. Ashton and al.,UK, 2002 | Intervention | Not podiatrist interventions |
| 1. Aydin and. Al., Turkey, 2010, | Intervention | Not podiatrist interventions |
| 1. Baba and al.,Australia, 2015 | Intervention | Not podiatrist interventions |
| 1. Bakker, UK, 2002 | Intervention | Not podiatrist interventions |
| 1. Baumeister and al., Germany, 2004 | Language | Article in German |
| 1. Berry and al., USA, 2004 | Outcomes/Intervention | Not podiatrist interventions/ not predeterminated outcomes |
| 1. Blatchfort, Austrlia, 2015 | Outcomes/Intervention | Not podiatrist interventions/ not predeterminated outcomes |
| 1. Bogedom and al., Netherlands, 2004 | Intervention | Not podiatrist interventions |
| 1. Branchaud and al., USA, 1998 | Intervention | Not podiatrist interventions |
| 1. Butter, New Zealand. 2011 | Population/Outcomes | Not podiatrist interventions/ not predeterminated outcomes |
| 1. Cheng and al., Australia, 2016 | Intervention | Not podiatrist interventions |
| 1. Cigna and al., Italy, 2014 | Intervention | Not podiatrist interventions |
| 1. Conte and al., USA, 2012 | Intervention | Not podiatrist interventions |
| 1. Creagh and al., UK, 2012 | Intervention | Exposition to podiatry unclear |
| 1. De Corrado and al., Italy, 2013 | Language | Article written in Italian |
| 1. DeNamur and al., USA, 2002 | Design | Cases review |
| 1. Driver and al., USA, 2010 | Design | Review article |
| 1. Gibbons and al., USA, 1993 | Intervention | Not podiatrist interventions |
| 1. Gök and al., Turkey, 2016 | Intervention | Not podiatrist interventions |
| 1. Ha Van, France, 2013 | Intervention | Not podiatrist interventions |
| 1. Hartmann and al., Germany, 2017 | Design/Intervention | Case series/ No control/comparison group |
| 1. Horswell and al., USA, 2003 | Intervention | Program/ Unclear podiatrist interventions |
| 1. Hsu and al., Taiwan, 2015 | Intervention | Not podiatrist interventions |
| 1. Idris and al., UK, 2005 | Intervention | Not podiatrist interventions |
| 1. Ignatyeva and al., Russia, 2015 | Article type | Conference abstract |
| 1. Jude and al., Greece, 2013 | Article type | Conference abstract |
| 1. Kalidinli and al., UK, 2016 | Article type | Conference abstract/audit |
| 1. Khandelwal and al., India, 2013 | Intervention | Not podiatrist interventions |
| 1. Knowles and al., UK, 1996 | Design | Narrative review |
| 1. Komelyagina and al., Russia, 2016 | Language | Article written in Russian |
| 1. Lauterbach and al., Germany, 2010 | Intervention | Not podiatrist interventions/ attending to podiatry practice for 1^st^ consultation/ no comparative group |
| 1. Lawrence and al., Australia, 2002 | Intervention | Not podiatrist interventions |
| 1. Lavery and al., USA, 2004 | Intervention | Not podiatrist interventions |
| 1. Liang and al., China, 2012 | Intervention | Not podiatrist interventions/ no podiatrist involved |
| 1. Litzelman and al., USA, 1993 | Intervention | Not podiatrist interventions/ no podiatrist involved |
| 1. Martinez-Gomez and al., Spain, 2014 | Intervention | Not podiatrist interventions/ no podiatrist involved |
| 1. McGill and al., Australia, 2005 | Intervention | Not podiatrist interventions |
| 1. McInnes and al., UK, 2009 | Design | Consensus statement for a framework on diabetic foot with podiatrist |
| 1. Moulik, UK, 2003 | Intervention | Not podiatrist interventions/data from podiatry |
| 1. Perrin and al., Australia, 2006 | Design | Audit |
| 1. Pityk and al., Ukraine, 2013 | Language | Article written in Ukrainian |
| 1. Rasmussen and al., Denmark, 2015 | Intervention | Not podiatrist interventions |
| 1. Rayman and al., UK, 2004 | Intervention | Not podiatrist interventions |
| 1. Reiber and al., USA, 2007 | Outcomes | Not predeterminated outcomes on DFUs and LEA |
| 1. Rijken and al., Netherlands, 1999 | Outcomes/Design | Not predeterminated outcomes on DFUs and LEA/cases series |
| 1. Rosati and al., Italy, 2012 | Design | Cases series |
| 1. Roth-Albin and al., Canada, 2017 | Intervention | Not podiatrist interventions |
| 1. Searle and al., UK, 2008 | Design | Narrative review |
| 1. Smith and al., UK, 2016 | Article type | Conference abstract |
| 1. Stanley and al., UK, 2004 | Design | Narrative review |
| 1. Thomas and al., UK, 2016 | Intervention | Not podiatrist interventions |
| 1. Traore and al., Ivory Coast, 2001 | Design | Cross-sectional study |
| 1. Wilbek and al., Denmark, 2016 | Intervention | Not podiatrist interventions/ no podiatrist involved |
| 1. Walker, UK, 2016 | Design | Case study of 5 patients |
| 1. Weck and al, Germany, 2007 | Language | Article written in German |
| 1. Wrobel and al., USA, 2006 | Design | Cross-sectional study |
| 1. Zhang and al., Australia, 2015 | Outcome | Not predeterminated outcomes on DFUs and LEA |
| 1. Baillie and al., UK, 2017 | Missing Data/Design | No association measure available, no description of population and n |
| 1. Patout and al., USA, 2000 | Intervention | Missing information about podiatrist intervention in the CD-LEAP group, not specify |
| 1. Meltzer and al., USA, 2002 | Design | Case-control study |
| 1. Hedetoft and al., Denmark 2008 | Design | Case-control study |
| 1. Plank and al., Austria, 2003 | Intervention | Podiatry without a team |
| 1. Lavery and al., UK, 2010 | Intervention | Podiatry without a team |
| 1. Schmidt and al., USA, 2017 | Reported elsewhere | Schmidt and al, 2018 |
| 1. Gibson and al., USA, 2013 | Intervention | Podiatry without a team |
| 1. Carls and al., USA, 2011 | Intervention | Podiatry without a team |
| 1. Lipscombe and al., Canada, 2003 | Intervention | Podiatry without a team |
| 1. Sowell and al., USA, 1999 | Intervention | Podiatry without a team |
| 1. Ronnemaa and al., Finland, 1997 | Intervention | Podiatry without a team |
| 1. Hamalainen and al., Finland, 1998 | Intervention | Podiatry without a team |
| 1. Roberts and al, Canada, 2019 | Article type | Conference abstract |
| 1. Paisey and al., UK, 2019 | Article type | Audit |
| 1. Bullen and al., UK, 2019 | Design | Cross-sectional study |
| 1. Paisey and al., UK, 2019 | Article Type | Audit |
| 1. Shapiro, Canada, 2020 | Design | Cross-sectional study |
| 1. Bhattacharyya, UK, 2019 | Article type | Conference abstract |
| 1. Bhandari and al., India, 2019 | Design | Cross-sectional study |
| 1. Fonseca and al., Portugal, 2019 | Article type | Conference abstract |
| 1. Saidel-Odes and.al, Middle East Jerusalem, 2019 | Article type | Conference abstract |
| 1. Riaz and al., Pakistan, 2019 | Intervention | Not podiatrist interventions/ no podiatrist involved |
| 1. Mungai and al., Kenya, 2019 | Intervention/Design | Not podiatrist interventions/Cross-sectional study |
| 1. Wang and al., China, 2019 | Design | Cross-sectional study |
| 1. Wennberg and al., Sweden, 2019 | Outcomes/Intervention | Not podiatrist interventions/ not predeterminated outcomes |
| 1. Musuuza and al., USA, 2019 | Design | Systematic review |
| 1. Brocco and al., Italy, 2018 | Design | Review |
| 1. MacRury and al., UK, 2018 | Outcomes/Intervention | Not podiatrist interventions/ not predeterminated outcomes |
| 1. Albright and al., USA, 2020 | Design | Systematic review |
| 1. Dutra and al., Brazil, 2019 | Outcomes/Intervention | Not podiatrist interventions/ not predeterminated outcomes |
| 1. Hicks and al., USA, 2019 | Intervention | Difference between intervention/podiatrist exposition and no control/comparison group |
| 1. Hicks and al., USA, 2019 | Intervention | Difference between intervention/podiatrist exposition and no control/comparison group |
| 1. Dalla Paola and al., Italy, 2019 | Intervention | Not podiatrist interventions |
| 1. Messenger and al., Kuwait, 2018 | Design | No control/comparison group |
| 1. McLean and al.,Australia, 2019 | Outcomes/Intervention | Not podiatrist interventions/ not predeterminated outcomes |
| 1. Forde and al., Ireland, 2019 | Outcomes/Intervention | Not podiatrist interventions/ not predeterminated outcomes |
| 1. Kim and al., Korea, 2019 | Intervention | Podiatry not clearly involved, intervention stated as podiatric surgeries |
| 1. Aalaa and al., Iran, 2019 | Article type | Commentary |
| 1. Perrin and al., Australia, 2019 | Outcomes/Intervention | Not podiatrist interventions/ not predeterminated outcomes |
| 1. Loke and al., Singapore, 2019 | Article type | Conference abstract |
| 1. Brennan and al., Netherland, 2019 | Article type | Conference abstract |
| 1. Fukuta and al., Japan, 2019 | Article type | Conference abstract |
| 1. Jiménez and al., Spain, 2018 | Intervention | Not podiatry intervention especially/No control group |
|  |  |  |
|  |  |  |
